# Cervical vestibular evoked myogenic potentials in 3-month-old infants: Comparative characteristics and feasibility for infant vestibular screening

**DOI:** 10.3389/fneur.2022.992392

**Published:** 2022-09-29

**Authors:** Jiali Shen, Lu Wang, Xiaobao Ma, Zichen Chen, Jianyong Chen, Xueyan Wang, Kuan He, Wei Wang, Jin Sun, Qin Zhang, Min Shen, Xiangping Chen, Qing Zhang, Kimitaka Kaga, Maoli Duan, Jun Yang, Yulian Jin

**Affiliations:** ^1^Department of Otorhinolaryngology-Head and Neck Surgery, Xinhua Hospital, Shanghai Jiaotong University School of Medicine, Shanghai, China; ^2^Shanghai Jiaotong University School of Medicine Ear Institute, Shanghai, China; ^3^Shanghai Key Laboratory of Translational Medicine on Ear and Nose Diseases, Shanghai, China; ^4^Department of Otorhinolaryngology-Head and Neck Surgery, Second Affiliated Hospital of Xi'an Jiaotong University School of Medicine, Xi'an, China; ^5^Department of Otolaryngology-Head and Neck Surgery, Yanbian University Hospital, Yanji, China; ^6^National Institute of Sensory Organs, National Hospital Organization Tokyo Medical Center, Tokyo, Japan; ^7^Ear Nose and Throat Patient Area, Trauma and Reparative Medicine Theme, Karolinska University Hospital, Stockholm, Sweden; ^8^Division of Ear, Nose, and Throat Diseases, Department of Clinical Science, Intervention and Technology, Karolinska Institutet, Stockholm, Sweden

**Keywords:** cervical vestibular evoked myogenic potentials, infant, vestibular screening, hearing, hearing loss, hearing screening

## Abstract

**Objective:**

We compared the characteristics of air-conducted sound cervical vestibular evoked myogenic potential (ACS-cVEMP) and bone-conducted vibration cVEMP (BCV-cVEMP) among 3-month-old infants with normal hearing and sensorineural hearing loss (SNHL), and healthy adults to explore the feasibility and optimal strategies for infant vestibular screening.

**Methods:**

29 infants (58 ears) were divided into two groups according to hearing (group I: normal hearing ears; group II: SNHL ears), 20 healthy adults were defined as group III. The results of response rate, P13 and N23 latency, P13-N23 interval, amplitudes, and corrected interaural asymmetry ratio (IAR) were recorded and compared among three groups.

**Results:**

The response rates of ACS-cVEMP in three groups were 88.89, 62.00, 100%, respectively. The P13 and N23 latencies, and P13-N23 interval did not differ significantly between group I and II (*p* = 0.866, *p* = 0.190, *p* = 0.252). A significant difference was found between group I and III (*p* = 0.016, *p* < 0.001, *p* < 0.001). No significant difference was observed in raw or corrected amplitude between group I and II (*p* = 0.741, *p* = 0.525), while raw and corrected amplitudes in group III were significantly larger than group I (*p* < 0.001, *p* < 0.001). For BCV-cVEMP, the response rates in three groups were 100, 86.36, 100%, respectively, No significant difference existed in the P13 and N23 latency, or P13-N23 interval between group I and II (*p* = 0.665, *p* = 0.925, *p* = 0.806), however, P13 and N23 latencies were significantly longer in group III than group I (*p* < 0.001, *p* = 0.018), but not in P13-N23 interval (*p* = 0.110). There was no significant difference in raw or corrected amplitude between group I and II (*p* = 0.771, *p* = 0.155) or in raw amplitude between group I and III (*p* = 0.093), however, a significant difference existed in corrected amplitude between group I and III (*p* < 0.001).

**Conclusions:**

Compared with adults, 3-month-old infants with normal hearing presented with equivalent response rates, shorter P13 and N23 latencies, smaller corrected amplitudes, and a wider IAR range for both ACS and BCV-cVEMP. SNHL infants had equivalent response rates of BCV-cVEMP, lower response rates of ACS-cVEMP than normal hearing infants. When responses were present, characteristics of ACS and BCV-cVEMP in SNHL infants were similar with normal hearing infants. ACS combined with BCV-cVEMP are recommended to improve the accuracy of vestibular screening.

## Introduction

The vestibular sensory organ plays a non-substitutable role in the balance control. The vestibular system begins to develop *in utero* earlier than cochlea, and its morphology is well differentiated on the 49th day of gestation (1–3). At birth, the vestibular nerves are completely myelinated, and the vestibular end organs are well-structured (2, 3).

Vestibular dysfunction leads to poor balance and delayed gross motor development (2–5). Furthermore, it causes detrimental influence on learning skills, mental health, and social emotional development as well (4–6). Therefore, early diagnosis and timely intervention are crucial to reduce adverse effects on all aspects (2–6).

The incidence of vestibular dysfunction in infants and young children ranges from 0.7 to 25% (7, 8). Several studies have shown that children with hearing loss are at a high risk of vestibular impairment, nearly 20–85% of children with sensorineural hearing loss (SNHL) having unilateral or bilateral vestibular dysfunction (9, 10). This wide range might be related to the different pathologies, the degree of hearing loss, the selection of the vestibular test and the diagnostic criteria (7–10). Angeli (11) reported that there were 20–70% infants who referred Universal Newborn Hearing Screening (UNHS) have vestibular disorders. The comorbidity of cochlear and vestibular impairment is likely related to the two organ's similar embryonic origin, approximate genetic basis of sensory epithelium, close anatomical structures, and same blood supply source. Therefore, they could be affected by the same genetic embryonic factors, drugs, pathogenic microbial infection, and environment (10, 12–16).

However, vestibular dysfunction in children is often underestimated or ignored due to their limited expressiveness for precisely described symptoms, and feasibility of vestibular tests (17–19). Vestibular assessment in pediatric is quite challenging, but it has gained increasing attention and interests in recent years. Given the importance and high incidence of vestibular dysfunction, the necessity and feasibility of vestibular screening naturally emerge.

At present, vestibular screening has not been widely performed due to several reasons: firstly, it is difficult for infants and younger children to actively cooperate with the vestibular assessments, resulting in extremely challenging evaluation process; Secondly, specific screening tools, target population for screening, and the screening time point are not unified yet; Thirdly, the maturity of the vestibular system varies at different developmental stages, and test results from infants and younger children cannot be directly compared with reference data from adults. Normal reference values matching with children remain scanty.

UNHS has been conducted worldwide, contributing to early detection/diagnosis, and subsequent rehabilitation for infants with hearing loss. The international consensus (ICON) (20) and Joint Committee on Infant Hearing (JCIH) (21) recommended that those who failed hearing screening should accept diagnostic audiological assessment before 3 months of age. Whether the vestibular screening could be performed combining with diagnostic hearing test at 3rd month after birth to save travel time and reduce unnecessary troubles such as repeated appointments is worth attention and discussion.

Cervical vestibular evoked myogenic potential (cVEMP) recorded from the contracted sternocleidomastoid muscle (SCMM) is an objective, non-invasive, timesaving, reproducible and well-tolerated evaluation method, which can be selected as a screening test to evaluate the otolith function in young children (22, 23). In terms of its evoked stimuli, air conducted sound (ACS), the most commonly used stimulus, is frequently used to elicit cVEMP. Chen et al. (24) performed ACS-cVEMP in 24 healthy newborns aged 2–5 days, and the response rate was 75%, indicating that the sacculocollic reflex pathway is well responsive at birth. Sheykholesami et al. (25) reported the morphology of ACS-cVEMP in infants aged 1–12 months was similar to adults. Erbek et al. (3) observed presented ACS-cVEMP from all 20 full-term healthy infants aged 5–24 weeks. All these studies imply that cVEMP can be elicited at an early age. However, ACS-cVEMP is not suitable for subjects with conductive hearing loss. In contrast, bone conducted vibration cVEMP (BCV-cVEMP) can bypass the middle ear, allowing to evaluate the saccule and inferior vestibular nerve pathway for subjects with middle ear pathology (10, 23, 26). Verrecchia et al. (15) implemented BCV-cVEMP in infants aged 1–6 months who referred for the 2nd hearing screening due to the failure of the 1st hearing screening or had high risk factors of hearing loss, and those who came for diagnostic hearing assessment. Their subjects included both normal hearing and SNHL infants, however, they were not grouped by hearing. Marten et al. (16) conducted BCV-cVEMP as a vestibular screening tool in 6-month-old infants with hearing loss from 2018 to 2020. The study was quite instructive and reemphasizes the importance of vestibular screening, however, lack of age-matched normal controls and specific normal reference values were not displayed in their study.

The purpose of this study is to investigate the characteristics of ACS-cVEMP and BCV-cVEMP in 3-month-old infants with normal hearing, same age infants with SNHL and healthy adults, and explore the feasibility and optimal strategies for infant vestibular screening at 3rd month after birth.

## Materials and methods

### Subjects

Twenty-nine full-term 3-month-old infants who failed the 2nd hearing screening and referred to the Diagnosis and Treatment Center of Hearing Impairment and Vertigo in Xinhua Hospital affiliated to Shanghai Jiao Tong University School of Medicine from May 2021 to March 2022 were enrolled in this study, including 14 males and 15 females. All of them accepted ACS-cVEMP without sedation and 23 of them completed BCV-cVEMP as well. Then all of them completed tympanogram, Distortion Product Otoacoustic Emission (DPOAE), click-evoked Auditory Brainstem Response (c-ABR) and Tone-Burst ABR (TB-ABR) at 500 and 1,000 Hz under sedation. Some infants also completed 2,000 and 4,000 Hz TB-ABR, Auditory Steady-State Response (ASSR), depending on the degree of hearing loss.

Twenty-nine infants (58 ears) were divided into two groups according to their hearing. Group I included 27 normal hearing ears. Criteria for normal hearing as followings: no family genetic history, hypoxia, jaundice, viral infection, and other risk factors for hearing loss. Normal tympanogram with single or twin peaks at 1,000 Hz, passed DPOAE (four points passed at least in the six selected frequencies), air conducted c-ABR threshold ≤30 dB nHL. Group II included 29 SNHL ears. Criteria for SNHL: normal tympanogram, referred DPOAE (<4 points passed in the six selected frequencies), elevated air c-ABR threshold (>30 dB nHL), air and bone-conducted c-ABR threshold gap within 10 dB nHL.

For comparison, 20 healthy young adults (8 males and 12 females) were recruited as Group III, aged from 21 to 33 years old, with an average age of 25.10 ± 4.53 years old. All of them had normal tympanogram, 250–8,000 Hz pure-tone threshold ≤20 dB HL, no history of middle ear pathology, vestibular or neurological disease.

All the infants' parents and healthy adults signed the informed consent.

### Methods

#### Instruments and recording parameters of cVEMP

ACS-cVEMP was recorded by the electrophysiological device (Neuropack MEB-9400, NIHON KOHDEN, Japan). Sound stimulus of TB-500 Hz (the rise/fall time = 1 ms, the plateau time = 2 ms) at 132 dB peSPL (105 dBnHL) was presented monaurally through a calibrated headphone TDH-39 at a rate of 5 Hz. BCV-cVEMP was performed using the Eclipse device (Interacoustics, Denmark). Bone-conducted stimulus of TB-500 Hz was delivered using a B81 bone vibrator on the mastoid at 129.5 dB FL (60 dBnHL), and the stimulus rate was 5.1 Hz.

For both ACS-cVEMP and BCV-cVEMP, a minimum of 50 sweeps were averaged, and at least repeated twice to verify the waveform repeatability. The electromyogram (EMG) signals were amplified and bandpass filtered between 10 and 3,000 Hz. The recording window was −20–80 ms.

#### cVEMP testing procedure

Infants were entirely awake and placed in a supine position on the bed during testing. The local skin was treated with 75% alcohol and scrubbed lightly before the electrode placement. For ACS-cVEMP, the two reference electrodes were positioned at the upper third of the bilateral SCMM, the active electrodes were placed on the medial end of the clavicle on both sides. For BCV-cVEMP, the reference electrodes were positioned at the upper third of the bilateral SCMM, with an active electrode put on suprasternal notch, and the ground electrode was placed on the forehead in both tests. Electrode impedance was <5 kΩ and interelectrode impedance was roughly equivalent.

One audiologist operated the software, another one turned infant's head to the opposite side and tried to make the chin touched the shoulder to keep SCMM fully contracted. A family member comforted the infant and gently pressed the infant's shoulder to keep it from lifting. Toys and videos were used to distract the infant's attention. At least two trials were recorded on each side to confirm the waveform repeatability.

The cVEMP test parameters and electrode placement on healthy adults were the same as infants, while they were asked to rotate their heads toward the shoulder in the supine position, keeping the SCMM activated and tense until a certain procedure stopped.

#### Amplitude correction

For ACS-cVEMP, EMG activity was monitored on the screen. The mean rectified EMG of 20 ms pre-stimulation was calculated automatically by the device. The raw amplitude was divided by the mean rectified EMG to obtain the corrected amplitude. For BCV-cVEMP, the recording device has a function of EMG scaling to obtain the corrected amplitude. EMG activity was maintained at least >20 μV (15, 16, 24).

#### Investigational parameters of cVEMP

Characteristics of P13 and N23 latencies, P13-N23 interval, raw and corrected P13-N23 amplitudes were recorded. Since the cVEMP amplitude is strongly related to the strength of SCMM contraction, the interaural asymmetry ratio (IAR) was calculated using the corrected amplitude to compensate the bilateral amplitude difference caused by uneven EMG activity.

IAR = (AL – AS)/(AL + AS) × 100%, where AL is the larger corrected amplitude, AS is the smaller corrected amplitude (22, 24, 26).

The mean + 2SD of each parameter in normal hearing infants defined as the upper normal limit. Absent response or value exceeding the normal range was considered as abnormal.

### Audiological assessment

All infants were sedated with Chloral Hydrate (50 mg/kg) for subsequent audiological assessment. Tympanogram was obtained by Interacoustics AT235H Middle Ear Analyzer (Interacoustics, Denmark). Single or twin peaks at 1,000 Hz probe tone was considered as a normal middle ear function (27, 28).

DPOAE and ABR were recorded by the same instrument as BCV-cVEMP (Interacoustics, Denmark). For DPOAE, primary tone stimulus intensities were set at L1 = 65 dB SPL and L2 = 55 dB SPL, and the primary tone frequency ratio (f2/f1) was 1.22. 1,000, 2,000, 3,000, 4,000, 6,000, and 8,000 Hz were selected as test frequencies. Less than four of above frequencies passed with SNR ≥ 6 dB was defined as the refer criterion (29, 30).

For diagnostic ABR test, the active electrode was positioned on the center of the forehead, the ground electrode was put on the nasal root, and the reference electrodes were placed at the mastoid on both sides. Sound stimulus of click/Tone Burst in alternating polarity was delivered using a calibrated ER-3A inserted earphone at a stimulation rate of 37.1 Hz. The B81 bone vibrator was put on the mastoid of the test side and the non-test ear was masked. The bandpass filtered between 100 and 3,000 Hz. The recording window was 0–20 ms. A minimum of 1,024 sweeps were averaged. The maximum output of the stimulus was 95 dB nHL for air-conducted ABR and 45 dB nHL for bone-conducted ABR. The initial c-ABR stimulus intensity was 70 dB nHL. The stimulus intensity was initially reduced in 20 dB steps if wave-V was recognized, and if no wave-V was obtained at 70 dB nHL, the stimulus level delivered at 90 dB nHL directly. The ABR threshold was defined as the lowest stimulus intensity at which wave-V was still identifiable and repeatable. Two waveforms of absent wave-V at 5 dB nHL below the threshold intensity were necessary. The test sequence was air-conducted click, 500 and 1,000 Hz TB-ABR, bone-conducted c-ABR in order. TB-ABR at 2,000 and 4,000 Hz, ASSR were performed if necessary.

### Statistical analyses

Data were analyzed using SPSS software 26.0 (IBM, Armonk, NY). Normal distribution was evaluated by the Shapiro-Wilk test. A comparison between groups was performed by independent *t*-test for parametric variables and Mann-Whitney *U*-test for non-parametric variables. The chi-square test or chi-square correction for continuity test was used to compare the response rate of ACS-cVEMP and BCV-cVEMP between Group I and II, and between Group I and III, respectively. Independent *t*-test or Non-parametric Mann-Whitney *U*-test was used to compare the P13 and N23 latencies, P13-N23 interval, and the raw and corrected amplitudes between groups, respectively. *p* < 0.05 was considered to be statistically significant.

## Results

### Subject characteristics

**Figure 1:**
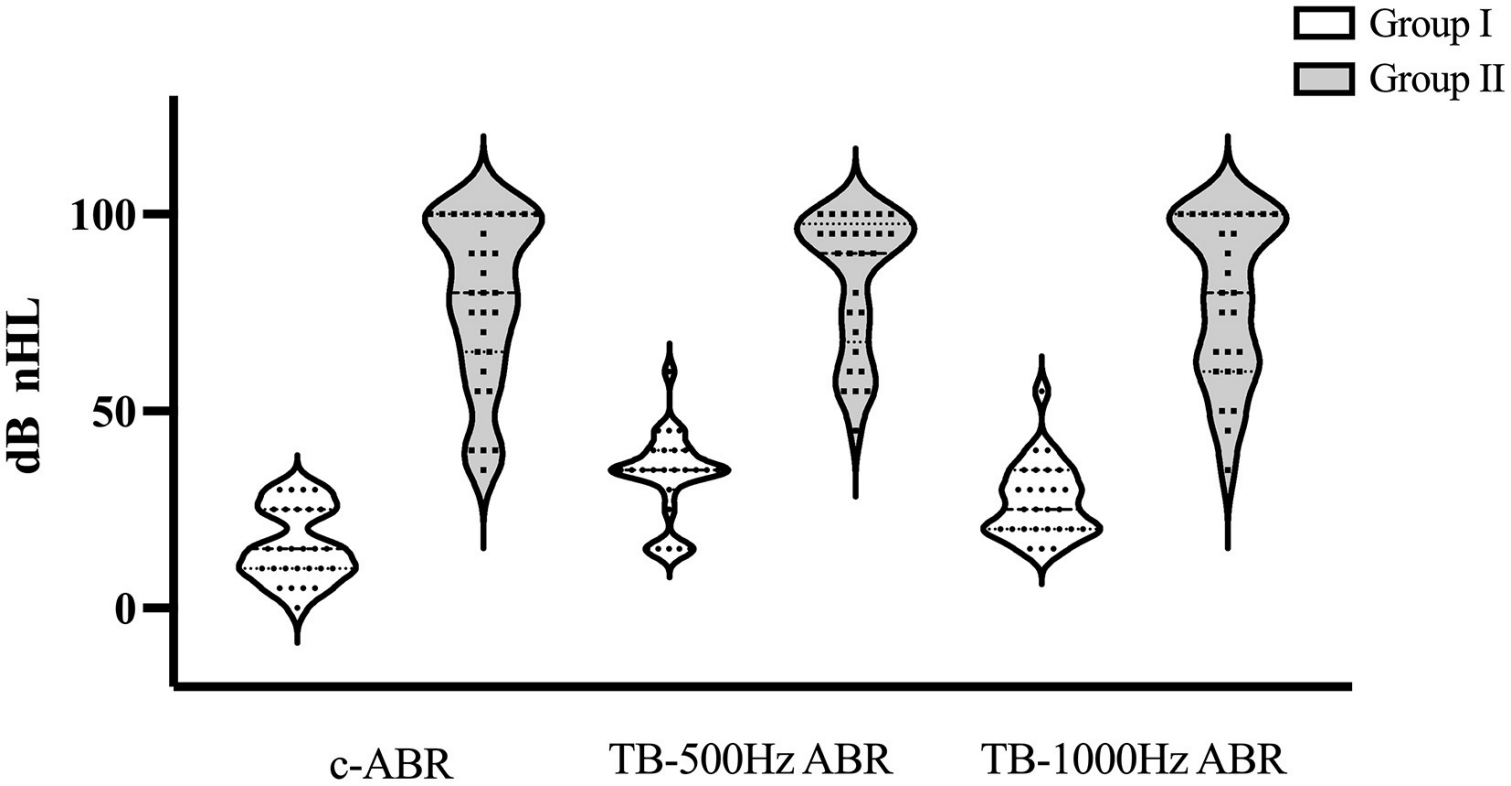
Hearing distributions in Group I and Group II. If there was no response at 95 dB nHL, a threshold of 100 dB nHLwas assumed. c-ABR, click-evoked Auditory Brainstem Response; TB-ABR, Tone-burst Auditory Brainstem Response.

Twenty-nine infants participated in this study, in which 12 infants had bilateral normal hearing, 14 infants had bilateral SNHL, and 3 infants had unilateral hearing loss. There were 2 ears with abnormal tympanogram in the unilateral hearing loss group which were excluded for ACS-cVEMP. Therefore, there were 27 normal hearing ears and 29 SNHL ears enrolled in ACS-cVEMP. Of these infants, 10 infants with bilateral normal hearing, 11 infants with bilateral SNHL and 2 infants with unilateral conductive hearing loss also completed BCV-cVEMP. On the whole, BCV-cVEMP was performed in 22 ears with normal hearing, 22 ears with SNHL and 2 ears with conducive hearing loss. Hearing distributions in Group I and Group II were depicted in Figure 1. The chi-square test showed there was no significant difference in gender among three groups of ACS-cVEMP or BCV-cVEMP (*p* = 0.550, 0.548, respectively, Table 1).

**Table 1:** Subjects characteristics in three groups.

	**ACS-cVEMP**			**BCV-cVEMP**		
	**Group I (*n* = 27)**	**Group II (*n* = 29)**	**Group III (*n* = 40)**	**Group I (*n* = 22)**	**Group II (*n* = 22)**	**Group III (*n* = 40)**
**Gender**
Male	11	16	20	10	14	20
Female	16	13	20	10	8	20
*p*	0.550			0.548		
χ^2^	1.195			1.204		

Group I: Normal hearing ears from infants; Group II: Sensorineural hearing loss ears from infants; Group III: Normal Hearing ears from adults.

n = number of ears.

Two ears (1 male and 1 female) with conductive hearing loss were not grouped.

The chi-square test showed there was no significant difference in gender among three groups of ACS-cVEMP or BCV-cVEMP (p = 0.550, 0.548, respectively).

### The waveform and response rate of ACS-cVEMP and BCV-cVEMP

**Figure 2:**
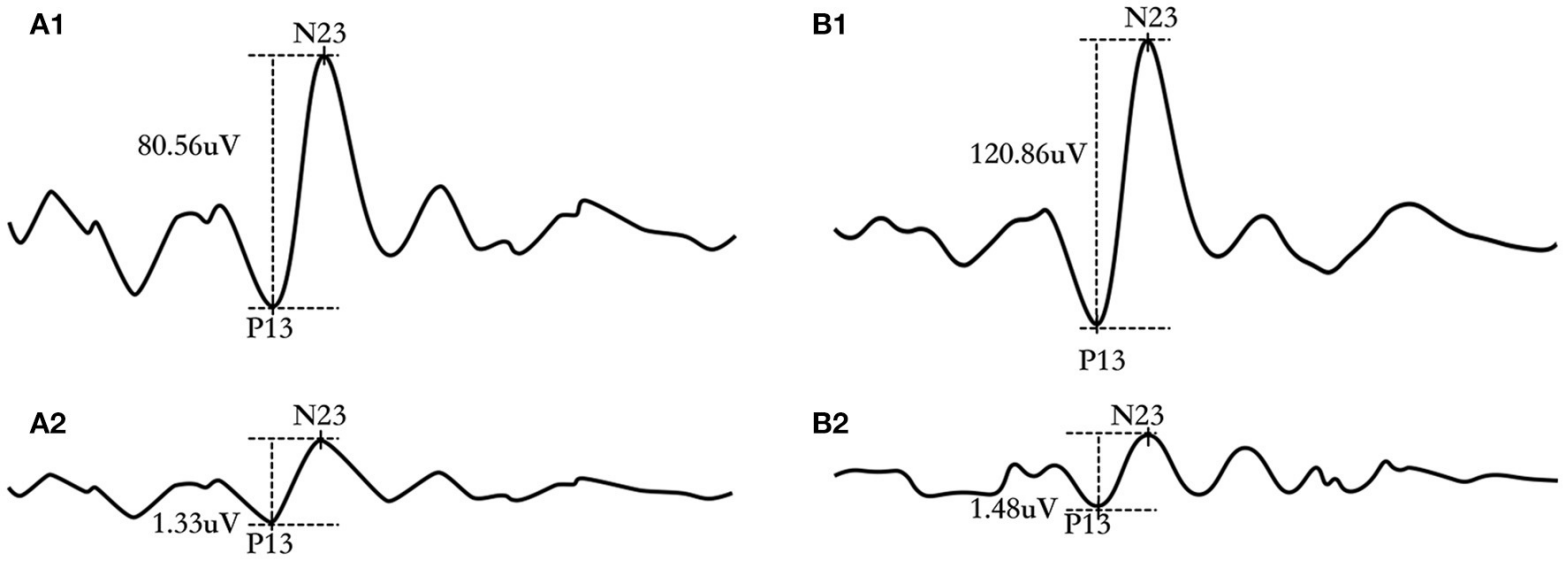
Representative ACS-cVEMP and BCV-cVEMP waveforms of a 3-month-old infant with bilateral normal hearing. **(A1)** raw waveform of ACS-cVEMP; **(A2)** corrected waveform of ACS-cVEMP; **(B1)** raw waveform of BCV-cVEMP; **(B2)** corrected waveform of BCV-cVEMP.

Figure 2 depicted raw and corrected ACS-cVEMP (Figures 2A1,A2) and BCV-cVEMP (Figures 2B1,B2) waveforms from a 3-month-old infant with normal hearing. P13 and N23 were marked at the initial positive and negative peak.

The response rate of ACS-cVEMP in three groups were 88.89, 62.00, and 100%, respectively (Table 2). A significantly lower response rate was found in group II than that in group I (*p* = 0.021), while there was no statistically significant difference between group I and group III (*p* = 0.120). The response rates of BCV-cVEMP in three groups were 100, 86.36, and 100%, respectively, in which there was no statistically significant difference between group I and II (*p* = 0.232), or between group I and III.

**Table 2:** The response rate of ACS-cVEMP and BCV-cVEMP in three groups.

**Group**	**Response rate**	
	**ACS-cVEMP**	**BCV-cVEMP**
I	88.89% (24/27)	100% (22/22)
II	62.00% (18/29)^a^	86.36% (19/22)^c^
III	100% (40/40)^b^	100% (40/40)

Group I: Normal hearing ears from infants; Group II: Sensorineural hearing loss ears from infants; Group III: Normal Hearing ears from adults.

a The chi-square test was used to compare the response rate of ACS-cVEMP between Group I and II. p^a^ = 0.021, χ^2^ = 5.364.

b The chi-square correction for continuity test was used to compare the response rate of ACS-cVEMP between Group I and III. p^b^ = 0.120, χ^2^ = 2.418.

c The chi-square correction for continuity test was used to compare the response rate of BCV-cVEMP between Group I and II. p^c^ = 0.232, χ^2^ = 1.431.

### P13 and N23 latencies and P13-N23 interval of ACS-cVEMP and BCV-cVEMP

**Figure 3:**
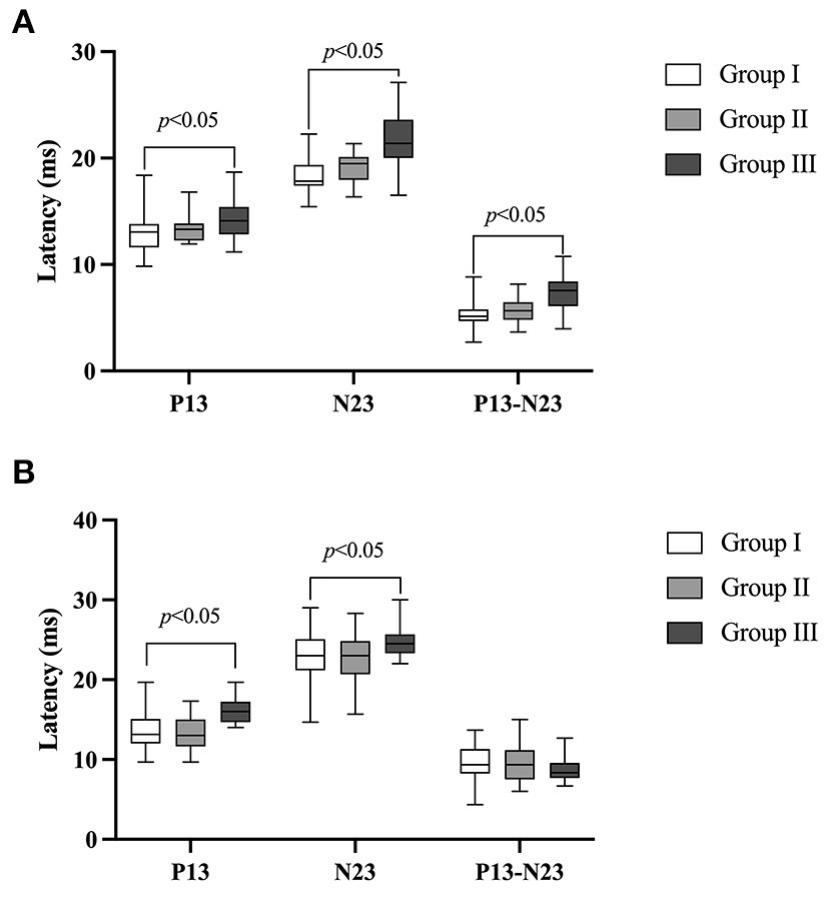
Comparison of the P13 and N23 latencies and P13-N23 interval of ACS-cVEMP and BCV-cVEMP. **(A)** Comparison of the P13 and N23 latencies and P13-N23 interval of ACS-cVEMP. The white box plot represented group I (*n* = 24 ears), light gray box plot represented group II (*n* = 18 ears), dark gray box plot represented group III (*n* = 40 ears). **(B)** Comparison of the P13 and N23 latencies and P13-N23 interval of BCV-cVEMP. The white box plot represented group I (*n* = 22 ears), light gray box plot represented group II (*n* = 21 ears), dark gray box plot represented group III (*n* = 40 ears). There was no significant difference of ACS-cVEMP in P13, N23 latency or P13-N23 interval between group I and group II. Significant differences were observed in all those parameters between groups I and group III. There was no significant difference of BCV-cVEMP in terms of P13 latency, N23 latency or P13-N23 interval between group I and group II. P13 and N23 latencies presented in Group III were significantly longer compared with group I, but not in the P13-N23 interval. In this study, no parameter comparison was made between group II and group III. Group I: Normal hearing ears from infants; Group II: Sensorineural hearing loss ears from infants; Group III: Normal Hearing ears from adults.

The descriptive data including mean, standard deviation (SD), median and interquartile range (IQR) of three groups were displayed in Table 3. The independent *t*-test revealed that the P13 and N23 latencies and P13-N23 interval of ACS-cVEMP did not differ significantly between group I and group II (*p* = 0.866, *p* = 0.190, *p* = 0.252, respectively Figure 3A). In contrast, statistically significant differences were found in these values between group I and group III (*p* = 0.016, *p* < 0.001, *p* < 0.001, respectively Figure 3A), indicating that significantly longer P13 and N23 latencies and P13-N23 interval presented in group III compared with group I.

**Table 3:** The P13 and N23 latencies and P13-N23 interval of ACS-cVEMP in three groups.

**Group**	***n* (ears)**	**P13 latency (ms)**				**N23 latency (ms)**				**P13-N23 interval (ms)**			
		**Mean**	**SD**	**Median**	**IQR**	**Mean**	**SD**	**Median**	**IQR**	**Mean**	**SD**	**Median**	**IQR**
I	24	13.13	1.90	13.08	11.61–13.80	18.40	1.85	17.83	17.43–19.36	5.27	1.20	5.13	4.68–5.79
II	18	13.21^a^	0.97	13.30	12.25–13.68	19.10^b^	1.40	19.48	17.96–20.10	5.73^c^	1.26	5.65	4.81–6.46
III	40	14.26^d^	1.69	14.10	12.83–15.43	21.63^e^	2.31	21.40	20.03–23.60	7.37^f^	1.70	7.55	6.10–8.40

Group I: Normal hearing ears from infants; Group II: Sensorineural hearing loss ears from infants; Group III: Normal Hearing ears from adults.

n = number of ears.

^a, b, c, d^Independent t-test was used to compare the P13 and N23 latencies and P13-N23 interval of ACS-cVEMP between Group I and II, and between Group I and III.

p^a^ = 0.866, t = 0.170; p^b^ = 0.190, t = 1.335; p^c^ = 0.252, t = 1.163.

p^d^ = 0.016, t = 2.474; p^e^ < 0.001, t = 5.816; p^f^ < 0.001, t = 5.283.

SD, Standard deviation; IQR, interquartile range.

The descriptive statistics of BCV-cVEMP in three groups were displayed in Table 4. The results indicated that there was no significant difference in the P13 latency, N23 latency or P13-N23 interval of BCV-cVEMP between group I and group II (*p* = 0.665*, p* = 0.925*, p* = 0.806, respectively Figure 3B). However, P13 and N23 latencies were significantly longer in group III than that in group I (*p* < 0.001, *p* = 0.018, respectively Figure 3B), but not in the P13-N23 interval (*p* = 0.110).

**Table 4:** The P13 and N23 latencies and P13-N23 interval of BCV-cVEMP in three groups.

**Group**	***n* (ears)**	**P13 latency (ms)**				**N23 latency (ms)**				**P13-N23 interval (ms)**			
		**Mean**	**SD**	**Median**	**IQR**	**Mean**	**SD**	**Median**	**IQR**	**Mean**	**SD**	**Median**	**IQR**
I	22	13.50	2.19	13.17	12.00–15.08	22.97	3.47	23.00	21.17–25.08	9.47	2.32	9.34	8.24–11.33
II	21	13.22^a^	1.98	13.00	11.67–15.00	22.87^b^	3.18	23.00	20.67–24.84	9.65^c^	2.51	9.34	7.51–11.17
III	40	16.11^d^	1.46	16.00	14.67–17.25	24.86^e^	1.92	24.50	23.33–25.67	8.74^f^	1.46	8.34	7.67–9.59

Group I: Normal hearing ears from infants; Group II: Sensorineural hearing loss ears from infants; Group III: Normal Hearing ears from adults.

n = number of ears.

^a, b, c, d^Independent t-test was used to compare the P13 and N23 latencies and P13-N23 interval of BCV-cVEMP between Group I and II, and P13 latency between Group I and III.

p^a^ = 0.665, t = 0.435; p^b^ = 0.925, t = 0.094; p^c^ = 0.806, t = 0.247; p^d^ < 0.001, t = 5.629.

^e, f^Mann-Whitney U-test was used to compare the N23 latency and P13-N23 interval of BCV-cVEMP between Group I and III. P^e^ = 0.018, z = 2.359; P^f^ = 0.110, z = 1.599.

SD, Standard deviation; IQR, interquartile range.

### Raw and corrected amplitudes of ACS-cVEMP

**Figure 4:**
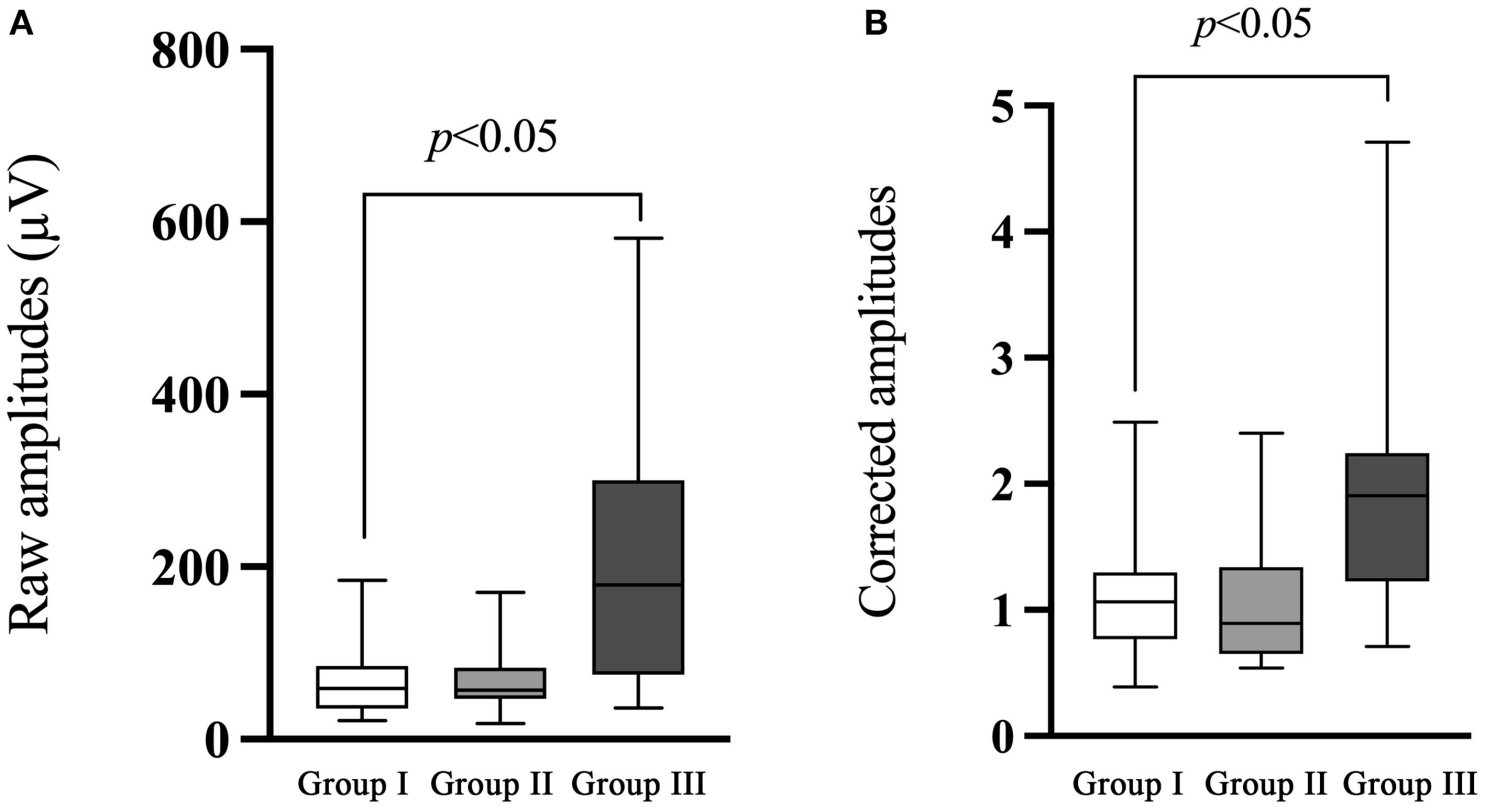
Comparison of the raw and corrected amplitudes of ACS-cVEMP. **(A)** The comparison of the raw amplitude of ACS-cVEMP. **(B)** The comparison of the corrected amplitude of ACS-cVEMP. The white box plot represented group I (*n* = 24 ears), light gray box plot represented group II (*n* = 18 ears), dark gray box plot represented group III (*n* = 40 ears). There was no significant difference in the raw or corrected amplitude between group I and group II. Group III had significantly larger raw and corrected amplitudes than that in group I. In this study, no parameter comparison was made between group II and group III. Group I: Normal hearing ears from infants; Group II: Sensorineural hearing loss ears from infants; Group III: Normal Hearing ears from adults.

The comparison of ACS-cVEMP between group I and group II demonstrated no significant difference in the raw or corrected amplitude (*p* = 0.741, *p* = 0.525, respectively, Table 5; Figure 4), while raw and corrected amplitudes in group III were significantly larger than that in group I (*p* < 0.001, *p* < 0.001, respectively, Table 5; Figure 4).

**Table 5:** The raw and corrected amplitudes of ACS-cVEMP in three groups.

**Group**	***n* (ears)**	**Raw amplitude (**μ**V)**				**Corrected amplitude (**μ**V)**			
		**Mean**	**SD**	**Median**	**IQR**	**Mean**	**SD**	**Median**	**IQR**
I	24	68.00	41.13	58.96	35.39–84.90	1.14	0.53	1.07	0.77–1.30
II	18	70.74^a^	40.36	57.18	46.73–83.00	1.07^b^	0.54	0.90	0.65–1.34
III	40	205.40^c^	138.97	179.26	74.94–300.63	1.93^d^	0.89	1.91	1.23–2.25

Group I: Normal hearing ears from infants; Group II: Sensorineural hearing loss ears from infants; Group III: Normal Hearing ears from adults.

n = number of ears.

^a, b, c, d^Mann-Whitney U-test was used to compare the raw and corrected amplitudes of ACS-cVEMP between Group I and II, and between Group I and III.

p^a^ = 0.741, z = 0.330; p^b^ = 0.525, z = 0.636; p^c^ < 0.001, z = 4.535; p^d^ < 0.001, z = 3.932.

SD, Standard deviation; IQR, interquartile range.

### Raw and corrected amplitudes of BCV-cVEMP

**Figure 5:**
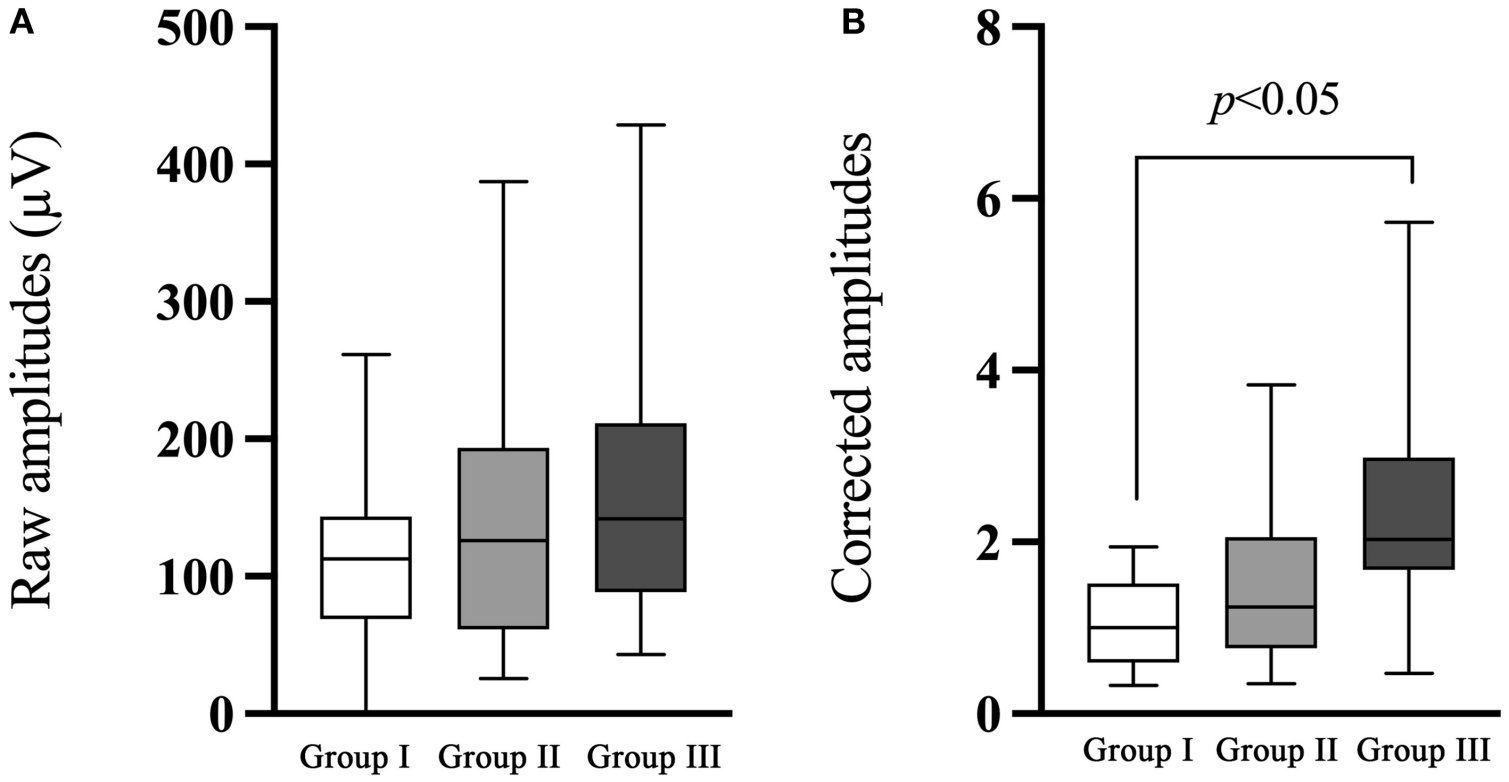
Comparison of the raw and corrected amplitudes of BCV-cVEMP. **(A)** The comparison of the raw amplitude of BCV-cVEMP. **(B)** The comparison of the corrected amplitude of BCV-cVEMP. The white box plot represented group I (*n* = 22 ears), light gray box plot represented group II (*n* = 21 ears), dark gray box plot represented group III (*n* = 40 ears). There was no significant difference of BCV-cVEMP in the raw or corrected amplitude between group I and group II, or in raw amplitude between group I and III. A significant difference existed in the corrected amplitude between group I and III. In this study, no parameter comparison was made between group II and group III. Group I: Normal hearing ears from infants; Group II: Sensorineural hearing loss ears from infants; Group III: Normal Hearing ears from adults.

There was no significant difference of BCV-cVEMP in the raw or corrected amplitude between group I and group II (*p* = 0.771, *p* = 0.155, respectively, Table 6; Figure 5). The raw amplitude was larger in group III compared with group I, but the difference did not reach statistical significance (*p* = 0.093). Significant difference existed between group I and group III with respect to the corrected amplitude of BCV-cVEMP (*p* < 0.001, Table 6; Figure 5).

**Table 6:** The raw and corrected amplitudes of BCV-cVEMP in three groups.

**Group**	***n* (ears)**	**Raw amplitude (**μ**V)**				**Corrected amplitude (**μ**V)**			
		**Mean**	**SD**	**Median**	**IQR**	**Mean**	**SD**	**Median**	**IQR**
I	22	124.69	61.59	114.55	73.85–148.73	1.04	0.52	1.00	0.53–1.54
II	21	143.49^a^	97.40	126.00	61.65–193.60	1.44^b^	0.88	1.24	0.77–2.06
III	40	162.69^c^	89.41	141.70	88.41–211.48	2.33^d^	1.05	2.03	1.67–2.98

Group I: Normal hearing ears from infants; Group II: Sensorineural hearing loss ears from infants; Group III: Normal Hearing ears from adults.

n = number of ears.

^a, b, c, d^Mann-Whitney U-test was used to compare the raw and corrected amplitudes of BCV-cVEMP between Group I and II, and between Group I and III.

p^a^ = 0.771, z = 0.292; p^b^ = 0.155, z = 1.422; p^c^ = 0.093, z = 1.677; p^d^ < 0.001, z = 5.017.

SD, Standard deviation; IQR, interquartile range.

### Corrected IAR of ACS-cVEMP and BCV-cVEMP

**Figure 6:**
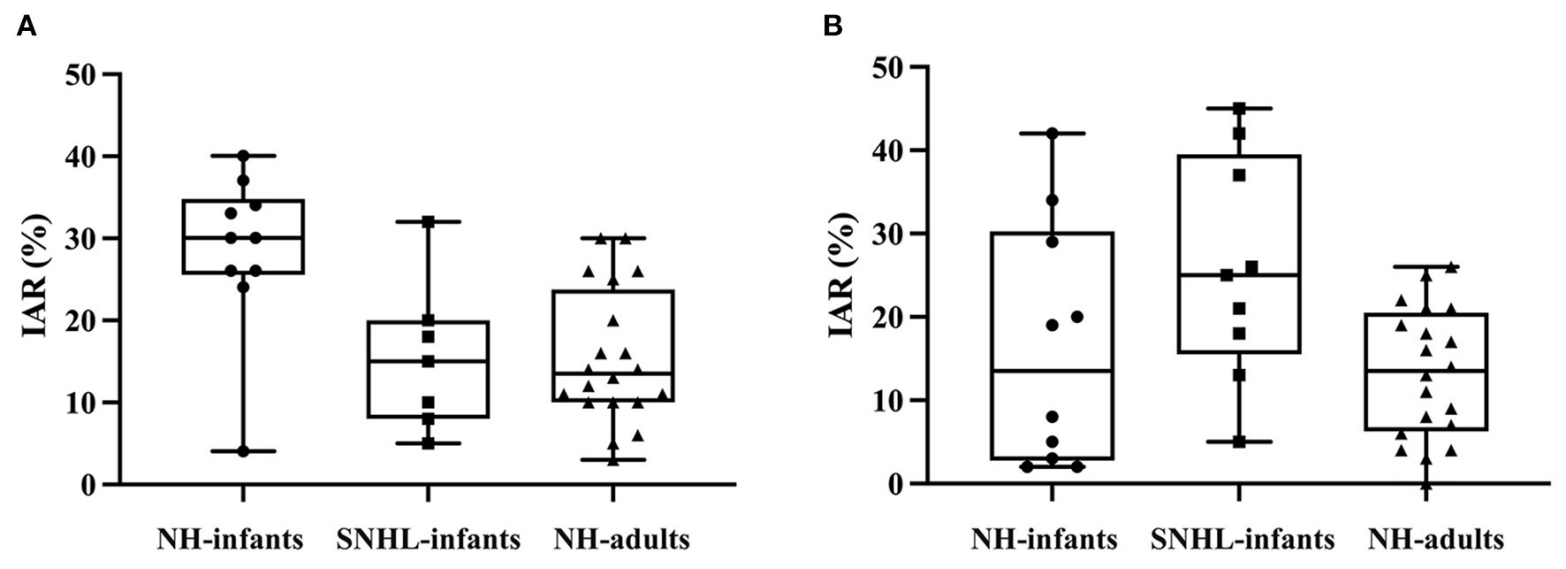
The distribution of corrected IAR of ACS-cVEMP and BCV-cVEMP. **(A)** The distribution of corrected IAR of ACS-cVEMP. **(B)** The distribution of corrected IAR of BCV-cVEMP. A wider range occurred in normal hearing infants compared with adults in both ACS-cVEMP and BCV-cVEMP. The corrected IAR of ACS-cVEMP in SNHL infants were fallen into the range of normal hearing infants. However, for BCV-cVEMP, the maximum corrected IAR of SNHL infants exceeded the maximum value of normal hearing infants. IAR: interaural asymmetry ratio. NH-infants: Normal hearing infants. SNHL-infants: Sensorineural hearing loss infants. NH-adults: Normal hearing adults.

The corrected IAR distribution in infants and adults were depicted in Figure 6. The corrected IAR of ACS-cVEMP had a median value of 30% in normal hearing infants (range: 4–40%, IQR: 25.50–34.75%), 15.00% in SNHL infants (range: 5–32%, IQR: 8.00–20.00%), and 13.50% in normal hearing adults (range: 3–30%, IQR: 10.00–23.75%). For BCV-cVEMP, the median values of corrected IAR were 13.50% in normal hearing infants (range: 2–42%, IQR: 2.75–30.25%), 25.00% in SNHL infants (range: 5–45%, IQR: 15.50–39.50%), and 13.50% in normal hearing adults (range: 0–26%, IQR: 6.25–20.50%).

The mean and SD of corrected IAR of ACS-cVEMP and BCV-cVEMP in normal hearing infants and adults who elicited cVEMP response bilaterally were shown in Table 7. Mean + 2SD was defined as the upper limit of normal values. The upper normal limit of IAR in normal hearing infants was larger than that in adults. The results showed that the corrected IAR ranges of ACS-cVEMP and BCV-cVEMP in infants with SNHL (5–32% in ACS-cVEMP, 5–45% in BCV-cVEMP) were within upper normal limit of infants with normal hearing.

**Table 7:** The Corrected IAR of ACS-cVEMP and BCV-cVEMP in group I and group III.

	**Corrected IAR of ACS-cVEMP (%)**				**Corrected IAR of BCV-cVEMP (%)**			
	**Mean**	**SD**	**Mean + 2SD**	***n***	**Mean**	**SD**	**Mean + 2SD**	***n***
Normal hearing infants	28.40	9.96	48.32	10	16.40	14.68	45.76	7
Normal hearing adults	15.40	8.15	31.70	20	13.20	7.85	28.90	20

n = number of subjects who elicited cVEMP response bilaterally.

IAR, interaural asymmetry ratio; SD, Standard deviation.

Mean + 2SD was defined as the upper limit of normal values.

## Discussion

Limited by the lack of B81 vibrator, unified test protocol, ACS-cVEMP is more accessible for most institutions compared with BCV-cVEMP at present. However, several studies have indicated BCV-cVEMP has the advantage of delivering sound directly to the inner ear and can be applied in infants and younger children who frequently present with conductive problems such as middle ear effusion, sebaceous glands and cerumen embolism in the external canal (1, 10, 15, 16). We performed both ACS-cVEMP and BCV-cVEMP, in order to provide normal reference values of these two stimuli modalities, and further promote the development of vestibular screening program.

### Comparison of cVEMP characteristics in infants and adults

To explore the maturation of sacculocollic reflex pathway and establish normal values for infants at the age of 3 months, we compared cVEMP characteristics between healthy adults and 3-month-old infants. Our results showed that the response rates of ACS-cVEMP and BCV-cVEMP did not differ significantly between ears from normal hearing infants and adults, indicating that the sacculocollic reflex has well developed at the 3rd month after birth, and its function can be evaluated by cVEMP reliably, which were consistent with the previous studies (25, 31, 32).

Shorter latencies in infants and children have been discovered in some previous studies (25, 33, 34). In the present study, we also observed significantly shorter P13 and N23 latencies in ears from infants than those from adults for both ACS-cVEMP and BCV-cVEMP (2, 25, 35–37). Authors reported that P13 and N23 latencies are highly correlated to the degree of myelinization and the length of the sacculocollic reflex pathway (2, 38, 39). Incomplete development and maturation of the vestibular reflex pathway would influence the nerve conduction velocity, resulting in prolonged latencies. Additionally, since the common embryonic origin of the saccule and cochlea, the delayed latency can also appear in the ABR test. It has been concluded that the vestibular system is fully developed and responsive at full-term birth, and the sacculocollic reflex pathway grows rapidly after birth (6, 39–42), however, the increased latencies mainly occur in preterm or neonates younger than 3 days as a result of hypomyelination. Our subjects were all 3-month-old full-term infants, and no prolonged latency presented during the ABR test. Therefore, we can safely assume that the maturation has no significant effect on latency in the current study. Moreover, studies reported the neck length can be used as an alternative way to estimate the pathway length, thereby a neck length of 15.3 cm as a cut-off point was proposed. There is a positive correlation between the neck length and cVEMP latency when it is within 15.3 cm. When exceeds this cut-off point, results are similar to that in adults (2, 43). Kelsch et al. (38) presented similar data, they found normal hearing children aged 3–5 years old had shorter latencies in comparison to those older than 5 years old, which is likely attributed to the increased path length with age.

In consideration of cVEMP amplitude, many studies have reported that smaller amplitude present in children compared with adults, which can be explained by the smaller muscle contraction in children (33). In agreement with previous studies, we also found a statistically significant smaller amplitude in ears from infants than that from adults. It has been well documented that EMG level is strongly correlated with cVEMP amplitudes (25). Raw amplitudes are less repeatable and present with wider variations due to the variability in SCMM contraction. Therefore, it is recommended that corrected amplitudes should be used if possible. Lee et al. (44) demonstrated that scaled amplitudes can provide more reliable and accurate information in the diagnosis of vestibular disorders. In this study, we monitored the EMG activity during the test procedure, and finally obtained the normalized values. As shown in Figures 4B, 5B, significantly larger corrected amplitudes of ACS-cVEMP and BCV-cVEMP were found in ears from adults than those from infants, which is probably due to the test conditions. Unlike adults, 3-month-old infants are unable to elevate or rotate their heads to contract the SCMM. As an alternative, one audiologist lifted infant's head and rotated it to the opposite side, during which infant may resist and cry, and the earphones and bone vibrator held by another audiologist may change position or slide, leading to smaller amplitudes. Consequently, it is of great importance to establish normal values in different age groups before using VEMP results for clinical diagnosis.

### Comparison of cVEMP characteristics in infants with normal hearing and SNHL

Many investigators have reported children with SNHL are at high risk of vestibular dysfunction, which could be explained by the close anatomical and embryological relationship between cochlea and vestibular end organs (45–48). Additionally, it has been reported that the etiology and degree of SNHL may be important predictors of vestibular dysfunction (45). Tribukait et al. (34) investigated vestibular function in children with profound hearing loss aged 15–17 years old indicating that the incidence of vestibular dysfunction was correlated with the degree of hearing loss, and it increased significantly when hearing loss worse than 90 dB nHL. Maes et al. (13) found that children with profound hearing loss had significantly higher abnormality rate of vestibular dysfunction than that in children with moderate hearing loss. Therefore, vestibular assessment of SNHL subjects is quite necessary.

While studies have demonstrated that cVEMP is a viable technique to evaluate the vestibular function in the pediatric population. Most of them targeted on children with vertigo symptoms, cochlear implant candidates, and SNHL children at an older age. Few studies included an age-matched normal controls, especially in infants, leading to a lack of normal reference values for comparison.

In the current study, we divided ears from infants into two groups by hearing. Our results showed that the response rate of ACS-cVEMP in SNHL ears was 62.00%, lower than that in normal hearing ears (88.89%). The results were agreement with previous studies (49, 50). For BCV-cVEMP, the response rate in ears with SNHL was 86.36%, which was in accordance with the recent studies (10, 16). Martens et al. (16) implemented BCV-cVEMP as a vestibular screening tool in 169 infants with hearing loss at the age of 6 months, nearly 88.8% infants passed the 1st screening, and 90.5% passed the 2nd screening at the age of 9 months. On the contrast, Verrecchia et al. (15) reported a higher BCV-cVEMP refer rate of 36.4% in children regardless of hearing. Verbecque et al. (9) demonstrated the refer rate of ACS-cVEMP in SNHL children was 43%. The various percentages of abnormalities may possibly relate to the following factors: Firstly, different characteristics of targeted subjects. Due to the close relationship between cochlea and vestibular organs, the etiology and degree of hearing loss play important role on the abnormal percentage. The study of Verrecchia et al. (15) included infants who had meningitis, fetal virus infections etc., which may result in a higher abnormal rate. Furthermore, the majority of the targeted population of Verbecque et al. (9) consisted of children with severe-profound hearing loss, while our study targeted at infants with different degree of hearing loss ranged from mild to profound. Thus, a higher risk of vestibular dysfunction may exist in their study. Secondly, test conditions are different. The stimulus modality (air conducted or bone vibration), intensity, and test position (supine or sit) are all related to the cVEMP results. Moreover, unequal diagnostic criteria in different institutions also lead to various interpretations (26, 51).

Interestingly, we observed there was no significant difference in terms of the response rate of BCV-cVEMP between normal hearing ears and SNHL ears, which was inconsistent in comparison with ACS-cVEMP. In addition to the individual-related and test-related influence factors which has been mentioned above, the response rate is highly related to the stimulus modality, which may also be used to explain the higher response rate of BCV-cVEMP than ACS-cVEMP in ears with SNHL in this study. Previous studies found lower response rate of ACS-cVEMP compared with BCV-cVEMP in adults (52–54). Taylor et al. (55) and Huang et al. (56) reported the abnormal prevalence of cVEMP elicited by ACS was higher than that of BCV in patients with Ménière's disease. On one hand, this may contribute to the different stimulus modality. Studies have reported that the mechanisms of ACS and BCV to activate otolith organs are different (57). It seems that ACS predominantly activates saccular afferents, while BCV stimulates both saccular and utricular afferent (58). Animal experiments have demonstrated that apart from the ipsilateral saccule pathways, otolith projections to the SCMM also include active potentials from the utricle (53, 58). Additionally, it has been shown that BCV stimulus can generate linear acceleration of the skull, while ACS stimulus only make labyrinth flow by pumping the stapes, so more otolith fibers are activated by BCV (41, 53, 58–60). Another hypothesis is that the hair cell cilia in the otolith deflect differently when stimulated by ACS and BCV. BCV induces more effective shear movement on the otolith membrane, leading to more hair cells activated (60). However, the exact mechanism is still being studied. On the other hand, it possibly caused by the limited number of subjects in our study, which should be further discussed in an enlarged sample size. In addition, the response rate of ACS-cVEMP is related with the middle ear status. The amniotic fluid and mesenchyme in the middle ear are not completely disappeared in newborns and infants. In our study, we performed 1,000 Hz tympanometry to assess the middle ear condition. However, some studies reported that although 1,000 Hz tympanometry is recommended to evaluate the middles ear status in infants under 6 months, it still has some limitations in terms of sensitivity and specificity (61, 62). Wideband tympanometry (WBT) has a wide range of stimulus from 226 to 8,000 Hz, which is more sensitive and could provide more informative data about the middle ear condition than traditional 226 or 1,000 Hz tympanometry. Studies indicated that WBT combined with previous medical history and otoscopy can improve the accuracy of middle ear function assessment (63–65). Therefore, the criterion of normal middle ear function in our study may not comprehensive enough, WBT should applied in further study.

In this study, characteristics of ACS-cVEMP and BCV-cVEMP in SNHL ears were similar with those in normal hearing ears. No significant difference was detected between two groups in P13 latency, N23 latency, P13-N23 interval, raw or corrected amplitude, which was in agreement with other studies. Maes et al. (13) investigated cVEMP in SNHL children aged 4–13 years old, demonstrating no significant difference existed in the above parameters when compared with normal-hearing peers. These findings may imply the response rate plays an important role in interpreting cVEMP results clinically.

### Corrected IAR of ACS-cVEMP and BCV-cVEMP

Previous studies recommended that amplitude normalization technique should be used during cVEMP test (66). Consequently, we mainly focused on the corrected IAR rather than raw IAR in different groups. Our results showed that the IAR range in normal hearing infants was broader than that in normal hearing adults in both ACS-cVEMP and BCV-cVEMP. And the IAR ranges of ACS-cVEMP and BCV-cVEMP in infants with SNHL were within the upper normal limit of normal hearing infants, implying that bilateral vestibular function is symmetrical in SNHL infants. Due to the small number of subjects in this study, it may not powerful enough to establish normal IAR reference values, further large-scale studies of IAR are required.

### Recommendations and strategies for vestibular screening in infants

Early detection of vestibular impairment can promote timely rehabilitation and reduce the negative impact on subsequent motor and balance development. In our study, clear and reproducible waveforms can be elicited by both ACS and BCV in normal hearing infants, and response rates were comparable to those of adults, indicating the feasibility of conducting cVEMP in infants at the age of 3 months. Additionally, it is recommended that infants who failed the 2nd hearing screening are expected to accept diagnostic hearing tests at the age of 3 months (20, 21). Based on these findings, our study performed cVEMP integrated with the clinical ABR diagnostic tests at 3 months of age. It was quite convenient as most ABR equipment includes VEMP module. In addition, it can avoid multiple round trips, reduce the number of appointments etc., which can contribute to a higher participate rate. Thus, we conclude that implementing the vestibular screening at 3rd months after birth may be appropriate and vestibular screening is technically feasible.

In terms of stimulus modality, majority of previous studies applied ACS in the vestibular assessment in infants and children. However, it should be noted that the response rate of ACS-cVEMP would be influenced by the conductive hearing loss which is common in pediatrics. BCV can bypass middle ear and suitable for subjects with middle ear pathology. However, limited by technology and cVEMP developmental maturity, not many institutions have access to the appropriate bone vibrator. Thus, it is meaningful to explore both ASC and BCV-cVEMP for extensive vestibular screening in different centers. Those who present with absent cVEMP are suggested to accept the 2nd screening at the age of 6 months to confirm the abnormality, which is also coincides with the 2nd diagnostic hearing loss tests and hearing-aid fitting if necessary. For centers equipped with bone vibrator, ACS-cVEMP combined with BCV-cVEMP are recommended in order to improve the accuracy of vestibular screening.

### Limitations

There are some limitations in this study should be noted. Firstly, not all subjects completed both ACS-cVEMP and BCV-cVEMP in this study, so there may be some deviations in subject selection that may affect the results. Secondly, limited by the number of infants in the current study, we did not discuss the effect of the degree or etiology of hearing loss on cVEMP characteristics, which should be further studied in a large sample scale. Thirdly, the devices for ACS-cVEMP and BCV-cVEMP were not unified. Furthermore, the specific passing criterion for vestibular screening needs to be further refined. And it should be noted that cVEMP does not reflect the canal function, a comprehensive evaluation is required in combination with other tests at an older age.

## Conclusion

According to this study, we draw a conclusion that ACS-cVEMP is feasible to evaluate vestibular function in infants at 3rd month after birth with a high response rate. ACS-cVEMP combined with BCV-cVEMP are recommended to improve the accuracy of vestibular screening, especially in those who have conductive middle ear problems. Early vestibular screening combined with hearing diagnosis is meaningful and worth of attention, which can minimize the negative effects on all aspects of life. Parameter values established in this study can provide references in clinical vestibular screening.

## Data availability statement

The original contributions presented in the study are included in the article/supplementary material, further inquiries can be directed to the corresponding authors.

## Ethics statement

The studies involving human participants were reviewed and approved by the Ethics Committee of Xinhua Hospital Affiliated to Shanghai Jiaotong University School of Medicine (Approval No. XHEC-D-2022-138). Written informed consent to participate in this study was provided by the participants' legal guardian/next of kin.

## Author contributions

JSh was responsible for data interpretation and manuscript preparation. LW and XW collected the clinical data. XM contributed to data analysis. ZC helped to optimize the cVEMP test procedure. KH, WW, JSu, and QinZ were responsible for auditory tests and cVEMP test assistance. JC, XC, and MS contributed to statistical consultation. QingZ, KK, and MD reviewed and revised the manuscript and study design. YJ and JY were responsible for the research design and manuscript revision. All authors contributed to the article and approved the submitted version.

## Funding

This work was funded by the National Natural Science Foundation of China (Nos. 81873698 and 81860189, 2019), and the Science and Technology Commission Foundation of Shanghai (No. 21Y31900504), and the Hospital Funded Clinical Research, Xin Hua Hospital Affiliated to Shanghai JiaoTong University School of Medicine, Clinical Research Unit (No. 21XHDB02, 2021).

## Conflict of interest

The authors declare that the research was conducted in the absence of any commercial or financial relationships that could be construed as a potential conflict of interest.

## Publisher's note

All claims expressed in this article are solely those of the authors and do not necessarily represent those of their affiliated organizations, or those of the publisher, the editors and the reviewers. Any product that may be evaluated in this article, or claim that may be made by its manufacturer, is not guaranteed or endorsed by the publisher.
